# Increasing *Sufu* gene dosage reveals its unorthodox role in promoting polydactyly and medulloblastoma tumorigenesis

**DOI:** 10.1172/jci.insight.176044

**Published:** 2024-02-15

**Authors:** Boang Han, Yu Wang, Shen Yue, Yun-hao Zhang, Lun Kuang, Bin-bin Gao, Yue Wang, Ziyu Zhang, Xiaohong Pu, Xin-fa Wang, Chi-chung Hui, Ting-ting Yu, Chen Liu, Steven Y. Cheng

**Affiliations:** 1 Department of Medical Genetics, Nanjing Medical University, Nanjing, China.; 2 Key Laboratory of Women’s Reproductive Health of Jiangxi, Jiangxi Maternal and Child Health Hospital, Nanchang, Jiangxi, China.; 3 Department of Pathology, Nanjing Drum Tower Hospital, Affiliated Hospital of Nanjing University Medical School, Nanjing, China.; 4 Department of Neurosurgery, Children’s Hospital of Nanjing Medical University, Nanjing, China.; 5 Program in Developmental and Stem Cell Biology, The Hospital for Sick Children, and Department of Molecular Genetics, University of Toronto, Ontario, Canada.

**Keywords:** Development, Oncology, Cancer, Mouse models

## Abstract

Suppressor of fused (SUFU) is widely regarded as a key negative regulator of the sonic hedgehog (SHH) morphogenic pathway and a known tumor suppressor of medulloblastoma (MB). However, we report here that SUFU expression was markedly increased in 75% of specimens compiled in a tissue array comprising 49 unstratified MBs. The SUFU and GLI1 expression levels in this MB array showed strong positive correlation, which was also identified in a large public data set containing 736 MBs. We further report that increasing *Sufu* gene dosage in mice caused preaxial polydactyly, which was associated with the expansion of the Gli3 domain in the anterior limb bud and heightened Shh signaling responses during embryonic development. Increasing *Sufu* gene dosage also led to accelerated cerebellar development and, when combined with ablation of the Shh receptor encoded by *Patched1* (*Ptch1*), promoted MB tumorigenesis. These data reveal multifaceted roles of *SUFU* in promoting MB tumorigenesis by enhancing SHH signaling. This revelation clarifies potentially counterintuitive clinical observation of high SUFU expression in MBs and may pave way for novel strategies to reduce or reverse MB progression.

## Abstract 2

Stabilization of GLI proteins by SUFU results in outsized SHH signaling responses, as manifested in altered limb development and medulloblastoma tumorigenesis.

## Graphical abstract

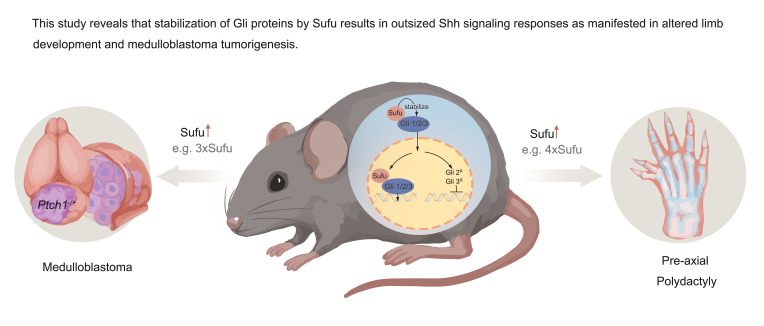

## Introduction

Medulloblastoma (MB) is a malignant brain tumor of the cerebellum that mostly affects children and young adults (1–3). Over the past two decades, large-scale genomic studies have revealed the presence of 4 molecular entities among MBs, consisting of wingless/Int (WNT), sonic hedgehog (SHH), group 3, and group 4 that can be stratified according to their distinct genomic, epigenomic, transcriptomic, or proteomic characteristics (3–8). Of these 4 groups, known driving causes of the SHH group MBs are powered by mutations in the SHH signaling pathway, *MYCN*, *TP53*, and *PTEN,* among others (9–15). However, the precise role of suppressor of fused (*SUFU*), one of the two well-recognized tumor suppressor genes in the SHH pathway, in addition to Patched1 (*PTCH1*), is not completely understood (5, 16–18). Previous studies indicated that genetically engineered *Sufu^–/+^* heterozygotic mice do not develop MBs unless the mutant allele was bred into the *p53* mutant background (19–21). Although these observations by themselves are not surprising, cerebellum-specific ablation of *Sufu* counterintuitively suppressed the tumor onset in mice initiated by the inactivation of *Ptch1* (18), which encodes a receptor of Shh (22). In fact, we reported previously that Sufu levels were elevated in spontaneously formed MBs in *Ptch1^–/+^* mice (17). Since *SUFU* status contributes to the gene signature that defines the newly revamped WHO molecular classification of MBs (23), a misunderstanding of its function may unnecessarily distort diagnostic assessment and adversely affect the treatment choices for this cancer.

SUFU is a core component of the SHH signaling pathway that guides pattern formation during embryonic development and controls the self-renewal of stem cells and tissue regeneration in adults (24–29). Biochemically, SUFU is a binding partner of 3 glioma-associated oncogene homolog (GLI) transcription factors that are executors of the SHH signaling responses (30, 31). The prevailing view of the SUFU mechanism is that it negatively regulates SHH signaling by acting as a cytoplasmic anchor of a sort to restrain downstream GLIs from traversing into the nucleus to initiate transcriptional responses (18, 32–35). However, we previously reported that mouse Sufu actually accompanies various Gli proteins shuttling into and out of the nucleus under the influence of the ligand, and the out-bound movement is influenced by a nuclear export signal, which is situated in an amorphous loop connecting the 2 globular domains in the Sufu molecular structure (30, 31) and is in juxtaposition to a dual phosphorylation site recognized by PKA and GSK3β (17, 36). The exact function and physiological significance of these phosphorylation events are not clear, but they may render Sufu more stable (36). Nevertheless, several reports suggest that this site is a key regulatory node of SUFU function in SHH signaling (37, 38). In addition to its transcriptional repressive activity, SUFU is also reported to possess the capacity to stabilize all 3 GLI proteins (39–41) and, by extension, to facilitate the generation of the proteolytically truncated GLI3 repressor, GLI3^R^, from full-length precursor that matures into the activator, GLI3^A^ (42, 43). This body of evidence prompted us to propose a potentially novel molecular chaperone model to explain SUFU function that accommodates both its negative and positive regulation of SHH signaling (17). In light of the intense effort in the field for searching downstream SHH pathway intervening nodes to circumvent the resistance to cell surface signal transducer SMO-based therapeutics (44–46), a proper mechanistic understanding of SUFU function is of great importance, given its central role in the pathway.

Here, we present evidence that SUFU levels in human MBs are also elevated and high levels of Sufu accelerated MB formation in mouse models with additional copies of human *SUFU* genes in the *Ptch1^–/+^* background. The additional gene copy allele was generated as a breeding intermediate in an attempt at addressing the regulatory function of the dual phosphorylation with insertions of human SUFU coding sequences containing altered S342 and S346 phosphorylation sites at the *Rosa26* locus. After combining with the “floxed” endogenous *Sufu^fl^* allele, we found that human SUFU cDNAs (hSUFUs) expressed from the *Rosa26* locus are fully capable of supporting embryonic development on their own, but mouse mutants with additional copies of *hSUFU* genes developed preaxial polydactyly and showed altered cerebellar development and, when crossed into the *Ptch1^–/+^* background, developed MBs with increased penetrance. These findings lend further support to the notion that SUFU is a potentially novel type of molecular chaperone with both tumor suppressing and promoting functions.

## Results

### Positive correlation between SUFU and GLI1 levels in human SHH MBs.

**Figure 1:**
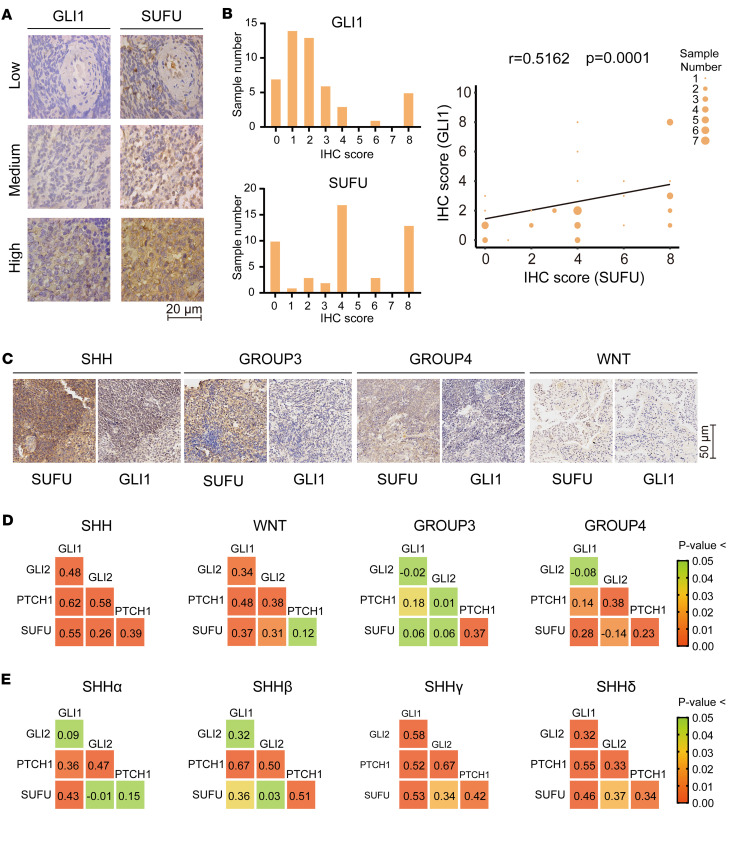
Positive correlation between GLI1 and SUFU expression in human MBs and SHH subgroup MBs. (**A**) Representative IHC staining of low, medium, and high SUFU and GLI1 expression in a MB tissue array. Note that 4 non-MB samples of total 53 specimens in the array were excluded from analysis. Scale bar: 20 μm. (**B**) IHC score distribution of GLI1 and SUFU staining and scatter plot of Pearson’s correlation between GLI1 and SUFU expression (**C**) IHC staining of SUFU and GLI1 in freshly resected and molecularly typed subgroups of human MB sections. For each MB group, *n* = 3. Scale bar: 50 μm. (**D**) Pearson’s correlation among relative SUFU, GLI1, GLI2, and PTCH1 levels in the new WHO MB grouping. The data were retrieved from a transcriptomic analysis of a public data set of 763 MBs deposited in the GEO database (GSE85217). (**E**) The same analysis as in **D** among 4 SHH MB subgroups. Two-tailed Student’s *t* test was used.

Elevated levels of Gli1 and Sufu in MBs that spontaneously developed in *Ptch1^–/+^* mice prompted us to determine if this was also the case in humans by examining GLI1 and SUFU expression in a tumor tissue array containing 49 unstratified MB specimens. Using a semiquantitative scoring matrix that took into account both IHC staining intensity and percentage of positively stained cells (47), we found that 75% of MB specimens showed medium to high SUFU expression (Figure 1, A and B, and Supplemental Table 1; supplemental material available online with this article; https://doi.org/10.1172/jci.insight.176044DS1). Likewise, close to 60% of cases showed medium and high levels of GLI1 expression, and a strong and statistically significant Pearson’s correlation was clearly present between SUFU and GLI1 (Figure 1, A and B, and Supplemental Table 1). Correlated high-level SUFU and GLI1 expression was also observed in freshly resected and molecularly typed human SHH MBs, but such correlation was low in WNT, group 3, and group 4 MBs (Figure 1C). Recently, similarity network fusion analyses of genome-wide gene expression and DNA-methylation data across 763 primary MBs revealed intertumoral heterogeneity within each MB group (3, 7). A reexamination of the RNA-Seq data set (GSE85217) from this large-scale study showed a strong Pearson’s correlation of high-level GLI1 and SUFU in the SHH group of MBs, less of a correlation in the WNT group and group 4, but no correlation in group 3 (Figure 1D). Intriguingly, similarity network fusion analysis assigned MBs with *SUFU* mutations to the SHHβ subgroup in which the correlation between levels of GLI1 and SUFU were the weakest among 4 different SHH subgroups (Figure 1E). This revelation is consistent with the notion that high level of SUFU is conducive to SHH signaling.

### Increased Sufu gene dosage leads to preaxial polydactyly in mice.

**Figure 2:**
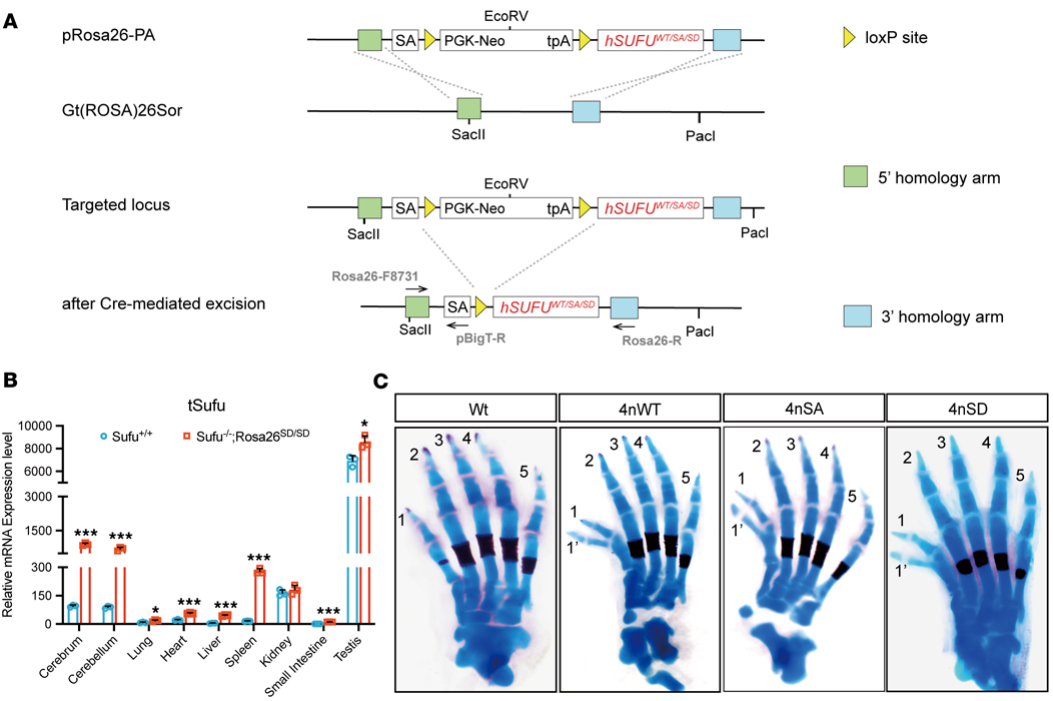
Construction strategy of *Sufu* gene dosage mutants and the preaxial polydactylous phenotype of 4nSD mice. (**A**) Schematic diagram of the homologous recombination targeting strategy for making Rosa26-transgenic lines. (**B**) Real-time qPCR quantification of total Sufu mRNA (tSufu) levels in various tissues of WT and *Sufu^–/–^; Rosa26^SD/SD^* mice. The PCR primer sequences used are common to both mouse *Sufu* and human *SUFU* gene transcripts. Data represent mean ± SD, and the 2-sided unpaired Student’s *t* test was used in the analysis. The experiment was repeated 3 times (*n* = 3). *P < 0.05, ***P < 0.001. (**C**) Alcian Blue/Alizarin Red staining showing additional toes in 4nWT (*n* = 4), 4nSA (*n* = 2), and 4nSD (*n* = 10) mice. Note the duplication of distal and intermediate phalanges of digit 1.

In an attempt at determining the physiological significance of dual phosphorylation of Sufu, we generated transgenic mice with hSUFU insertions at *Rosa26* locus via the pBigT vector (48, 49) and activated the inserted genes by *Ddx4*-Cre-mediated excision of the PGK-neo selection marker (Figure 2A). These transgenic lines were then combined with the *Sufu^fl^* allele, and the genotype distributions of Sufu coding sequences contributed by various copies of either endogenous or inserted alleles all followed Mendelian inheritance among offspring derived from intraline self-crosses, regardless of whether the inserted hSUFU contained serine-to–aspartic acid (SD) or serine-to-alanine (SA) changes at positions 342 and 346 or was the WT control (Supplemental Table 2 and Supplemental Figure 1). In mutants that contained only 2 copies of the inserted human *SUFU^SD^* genes at *Rosa26* loci (*Sufu^–/–^;* *Rosa26^SD/SD^*), the tissue distribution of the exogenous SUFU was similar to that of the endogenous one in WT mice, albeit the expression was at higher levels (Figure 2B). The mutant mice with 1–4 copies of *Sufu* genes all developed to term, but increasing gene dosage generated preaxial duplication of digit 1 with varying degrees of penetrance, depending on gene copies and sequences at the 342/346 positions (Figure 2C and Supplemental Table 3). In general, having 1 extra copy of *SUFU* gene was sufficient to generate preaxial polydactyly, but the SD mutant was much more potent than the SA mutant in doing so, possibly because the phosphorylation-mimicking SD mutant is more stable than the SA mutant (36). In the most severe and representative case of homozygotic insertion of the *SD* allele over the WT endogenous *Sufu* background (*Sufu^+/+^; Rosa26^SD/SD^*, henceforth named as 4nSD, with 4n denoting the total number of *Sufu* gene doses and SD the identity of contributing human *SUFU* mutant; the same nomenclature system is used for the SA and WT insertions), the polydactylous phenotype was seen in all limbs of 76.3% offspring (Supplemental Table 3). The penetrance of preaxial polydactyly in other mutants with 4 copies of *Sufu* genes, namely 4nSA and 4nWT, was lower but nevertheless substantial (Supplemental Table 3). Except for the limb phenotype, all copy number mutants appeared otherwise grossly normal and healthy in their daily activities. Since the mouse Sufu protein sequence is identical to that of human one and its functions is essential for embryonic development, the normal gross anatomy of *Sufu^–/–^* mice with *hSUFU* insertions indicates that the exogenous hSUFU expressed from Rosa26 locus is functionally indistinguishable from the endogenous mouse protein. Moreover, although the mutant mice with SD insertions exhibited higher polydactyly penetrance than their SA or WT counterparts, the differences appeared to be incremental, implying that the dual phosphorylation does not affect the patterning function of Sufu in limb development.

### Heightened Shh signaling accounts for the polydactyly associated with increased Sufu gene dosage.

**Figure 3:**
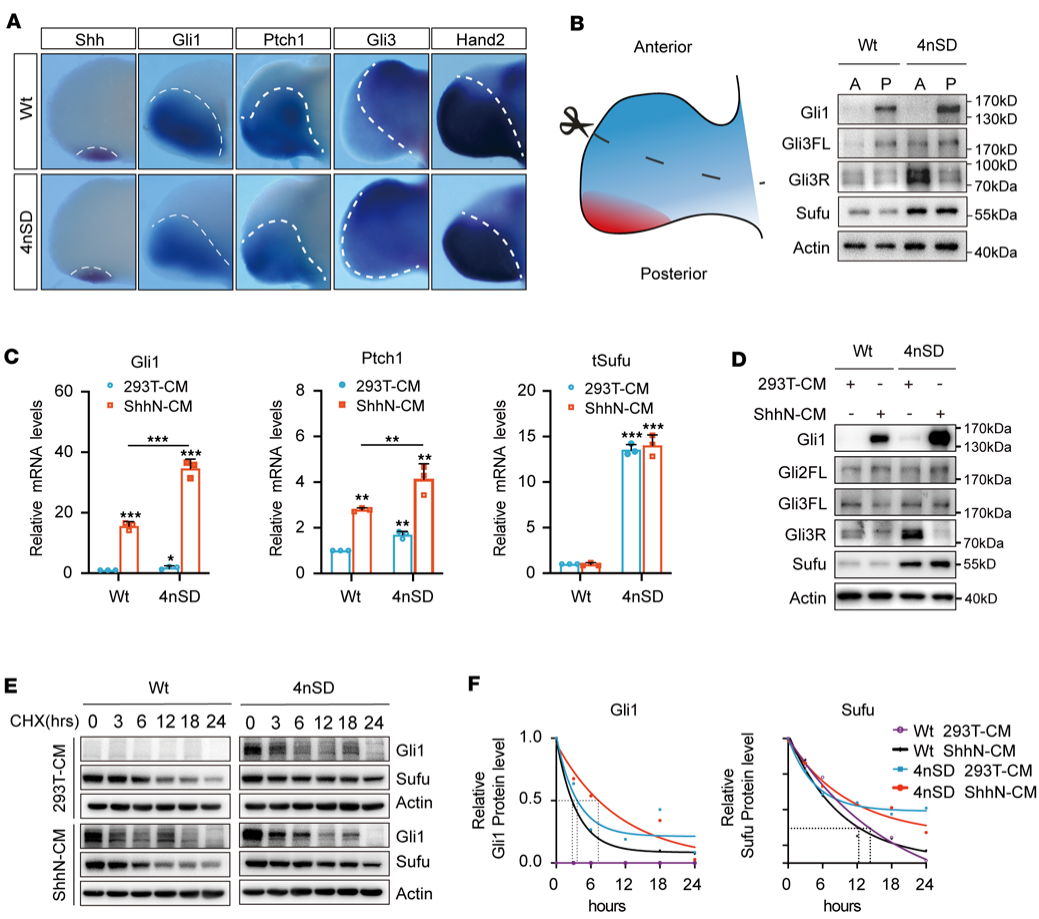
Preaxial polydactyly in 4nSD mice is associated with heightened Shh signaling. (**A**) RNA in situ hybridization analysis in E11.5 hind limb buds for *Shh* (*n* = 3), *Gli1* (*n* = 6), *Ptch1* (*n* = 6), *Gli3* (*n* = 5), and *Hand2* (*n* = 2). (**B**) Western analysis of protein expression in hind limbs of E11.5 mouse embryos. After dissection and cleaning, the limb buds were cut in the middle to separate anterior and posterior regions. The Sufu signal here encompasses both mouse and human proteins, as the antibody used reacts to both. Tissues from 4 limb buds were pooled for each sample analyzed. (**C**) Real-time qPCR analysis in MEFs freshly isolated from 4nSD or WT control embryos. The cells were treated with ShhN or control medium for 24 hours to activate signaling responses. The experiment was repeated 3 times (*n* = 3). Data represent mean ± SD, and 1-way ANOVA with Dunnett’s post hoc test was used for statistical analysis. (**D**) Western analysis in MEFs as described in **C**; the experiment was repeated 4 times (*n* = 4). (**E**) Western analysis of protein turnover and (**F**) quantification thereof in MEFs. CHX treatment was initiated 24 hours after signaling activation with conditioned mediums as above. The experiment was repeated 2 times (*n* = 2), and data represent mean ± SD. 2-way ANOVA with Bonferroni’s post hoc test was used for statistical analysis. *P < 0.05; **P < 0.01; ***P < 0.001.

To assess how increased *Sufu* gene dosage influences Shh signaling, we performed whole-mount in situ hybridization. The results showed normal expression of Shh in 4nSD limb buds at the zone of polarizing activity (ZPA), but the Gli1 and Ptch1 expression domains contracted posteriorly (Figure 3A). In contrast, Gli3 domain expanded along with a concomitant contraction of Hand2, a key posterior-anterior position cue of digit patterning that counteracts Gli3 (50–52). Consistent with the expansion of *Gli3* expression domain, increased accumulation of Gli3 proteins, both the full-length Gli3^A^ and truncated Gli3^R^, was detected by Western analysis in the anterior region of 4nSD limb buds (Figure 3B). However, it is intriguing to note that the level of Gli1, the target of Shh, also increased in the 4nSD posterior limb bud region and as did that of full-length Gli3^A^ (Figure 3B). These data suggest that a high level of Shh signaling was sustained cell autonomously in receiving cells as the Shh ligand was normally expressed in its usual domain (Figure 3A). Indeed, direct testing of signaling responses in freshly isolated murine embryonic fibroblasts (MEFs) from 4nSD and WT control mice showed that doubling *Sufu* gene dosage allowed more robust Shh induction of the pathway targets Gli1 and Ptch1 (Figure 3, C and D), while measurement of protein turnover rates showed stabilization of Gli1 in 4nSD MEFs (Figure 3, E and F). Similar enhancement of Shh signaling and stabilization of all forms of Gli factors was observed in 4nWT and 4nSA MEFs as well (Supplemental Figure 2). Taken together, the above observations demonstrated that elevated levels of Sufu allowed accumulation of more Glis, which in turn led to higher levels of Shh signaling in the posterior and stronger Gli3^R^ activities in the anterior limb bud regions.

### A high level of Sufu accelerates the onset of cerebellar development.

**Figure 4:**
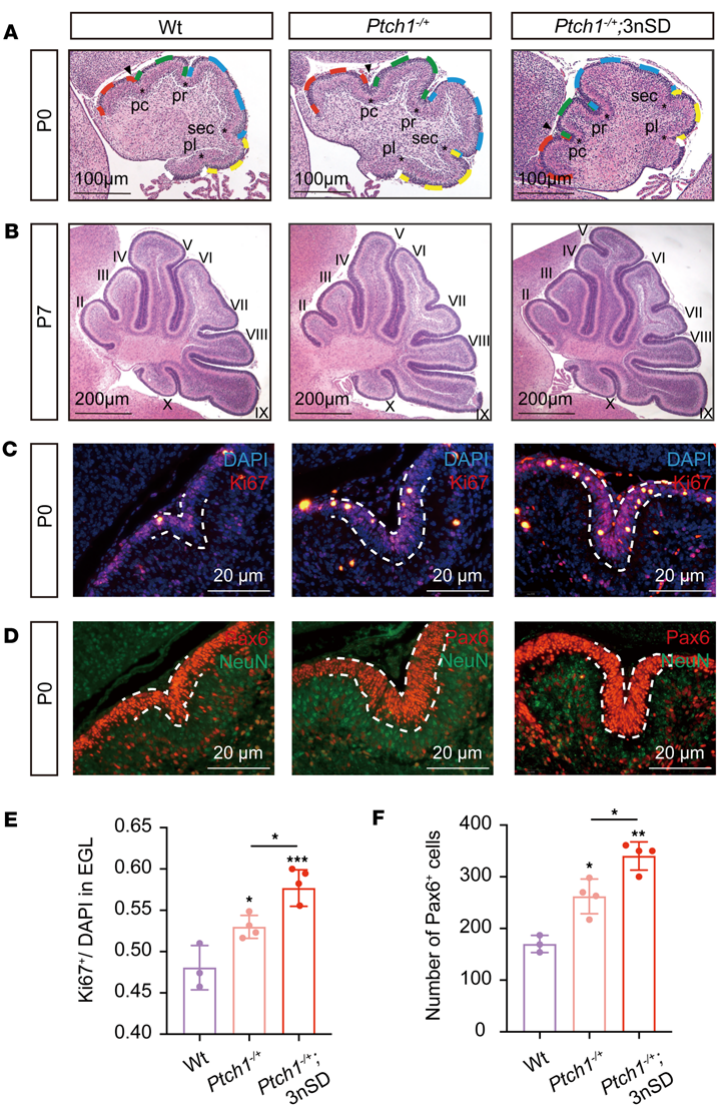
The initiation of cerebellar development is accelerated by high levels of Sufu. (**A** and **B**) H&E staining of sagittal midline cerebellar sections (**A**) at P0 (scale bar: 100 μm) and (**B**) at P7 (scale bar: 200 μm). For P0 cerebellar anlages, anterobasal (red), anterodorsal (green), central (blue), posterior (yellow), and inferior (white) cardinal lobes are marked with dash lines in their respective color. Note that the 4 principal fissures at the vermis of *Ptch1^–/+^;*3nSD cerebella were deeper than those of *Ptch1^–/+^* or WT ones. Mature cerebellar lobules are marked by Roman numerals, and principal fissures are marked by asterisks. pc, preculminate; pr, primary; sec, secondary; pl, posterolateral fissure (67). For each mouse line, 4 newborn pups were analyzed (*n* = 4). (**C**) Anti-Ki67 and (**D**) anti-NeuN immunofluorescence staining of P0 cerebellar sections at the pc fissure are shown with DAPI or anti-NeuN as counterstaining, respectively (scale bar: 20 μm). Combined images from both channels are shown, but the cells were counted in individual channels. (**E**) Ratio of proliferating GCPs (Ki67^+^ cells) and (**F**) total number of GCPs (Pax6^+^ cells) in a fixed EGL area surrounding the pc fissure (circled by the dash line). For each data point, 1 cerebellar section per mouse was stained and quantified, and total numbers of animals used were 3 for WT or 4 for *Ptch1^–/+^* and *Ptch1^–/+^;*3nSD mice, respectively. Data represent mean ± SD, and the 1-way ANOVA with Tukey’s multiple comparisons test was used for statistical analysis in **E** and **F**. *P < 0.05; **P < 0.01; ***P < 0.001.

In light of our previous demonstration that cerebellum-specific ablation of *Sufu* abolished *Ptch1* loss–induced MB tumorigenesis (18), the fact that high-level Sufu enhances Shh signaling in vivo raised an intriguing possibility that it may promote MB formation. To test this hypothesis, we introduced the *Ptch1^–^* allele into 4nSD mice and monitored cerebellar development and MB incidence. Although specified in mid gestation period, the cerebellar anlage does not become populated with differentiated neuronal cells until after birth. Shh signaling is absolutely essential to drive the proliferation of granule cell precursors (GCPs), which ultimately become the most abundant cell type in the cerebellum. In mice, the postnatal cerebellar development lasts over a period of 1 month, with the peak external germinal layer (EGL) expansion and cortex foliation during the first 2 weeks (53, 54). In WT mice, the foliation was barely visible in the cerebellar anlage at P0, but, in *Ptch1^–/+^* mice, this process was well advanced, as shown by the deepening of each of the 4 cardinal fissures (Figure 4A). In mice with 1 additional human *SUFU* gene dose (referred to as *Ptch1^–/+^;*3nSD mice), the foliation progressed even further at birth (Figure 4A). These observations imply that increasing *Sufu* gene dosage also enhanced Shh signaling induced by the partial loss of *Ptch1*. From P7 onward, the cerebellar development began to return to normal in *Ptch1^–/+^* mice (Figure 4B). To examine cerebellar development, we analyzed cerebella sections with immunofluorescence (IF) staining and focused on the preculminate fissure (pc) at the vermis to approximate the entire cerebellum (Figure 4, C and D). Quantification of the staining showed increased ratios of Ki67^+^ proliferating cells to DAPI decorated total number of cells in the EGL and increased total number of Pax6^+^ GCP cells at a given area surrounding the pc fissure in *Ptch1^–/+^* or *Ptch1^–/+^;*3nSD mice when compared with those of the WT mice at P0 (Figure 4, E and F). Similar acceleration of cerebellar development at birth was also observed in the tumor-free 4nSD mice compared with their matching WT littermates, which led to the broadening of average EGL width at P7 (Supplemental Figure 3). These data reaffirmed the notion that high-level Sufu enhances Shh signaling, which in turn leads to accelerated cerebellar development. However, high-level Sufu alone does not cause permanent damage to cerebellar development, as by P28 4nSD cerebella were indistinguishable from those of the WT controls (Supplemental Figure 3, J and K), and no visible anomaly was observed in the motor activities of 4nSD mice, even though these mice were preaxial polydactyly.

### High-level Sufu accelerates MB tumorigenesis.

**Figure 5:**
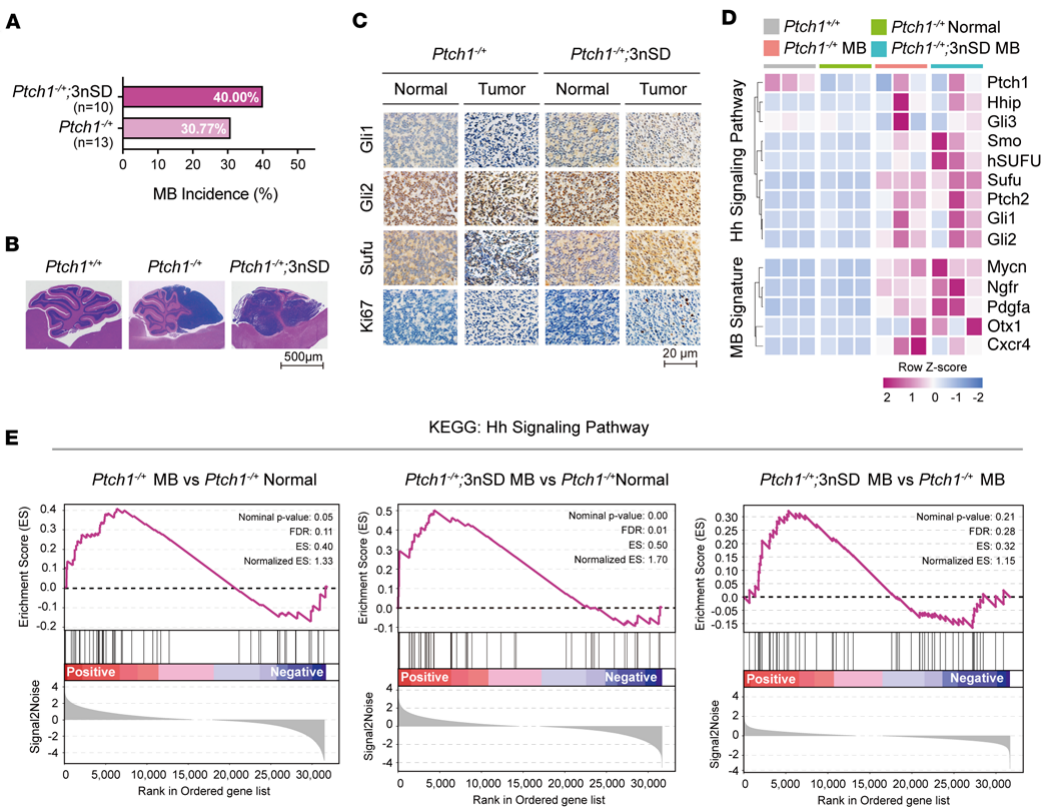
Increased *Sufu* gene dosage promotes MB tumorigenesis. (**A**) MB incidence in mice with the *Ptch1^–^* allele and different copies of *Sufu* genes. (**B**) H&E staining of normal cerebellum in WT mice (*n* = 3) and MBs in *Ptch1^–/+^* (*n* = 4) as well as *Ptch1^–/+^;*3nSD (*n* = 4) mice. Scale bar: 500 μm. (**C**) IHC comparison of marker gene expression between normal (peritumor) and MB tumor tissues in *Ptch1^–/+^* versus Ptch1^–/+^;3nSD mice. Three animals were analyzed in each mouse line. Scale bar: 20 μm. (**D**) Heatmap of transcriptomic profiling of Shh pathway components (*Hhip*, *Ptch1*, *Gli1*, *Gli2*, and *Gli3*) and MB hallmark genes (*Ngfr*, *Otx1*, *Cxcr4*, *Mycn*, and *Pdgfa*). Normal cerebellar as well as MB tumor tissues from 3 animals of each mouse line were used as biological repeats (*n* = 3) in the RNA-Seq analysis. (**E**) Enrichment analysis plots of the KEGG hedgehog signaling gene set, comparing expression in tumor versus normal tissues in 2 tumor models as well as comparing expression in 2 different tumor tissues.

As reported, the penetrance of spontaneous MBs in *Ptch1^–/+^* mice is about 30% (55). Compared with littermate controls, the addition of 1 extra copy of the *hSUFU^SD^* gene increased the MB penetrance to 40% at 6 months of age (Figure 5A), and the *Ptch1^–/+^;*3nSD MB tumor tissues appeared to have taken over the entire cerebellar volume (Figure 5B). It is noteworthy that we only obtained 2 *Ptch1^–/+^;*4nSD pups of 10 crosses, with 1 of the surviving pups developed MB. The lethality of *Ptch1^–/+^;*4nSD embryos was likely due to the inability of the neural tube to close (Supplemental Figure 4), another phenotype associated with abnormally heightened Shh signaling (20, 55). Consistent with the increased penetrance and severity, the levels of Gli1, Gli2, and Sufu expression were higher in *Ptch1^–/+^;*3nSD MB tumor tissues compared with those of the *Ptch1^–/+^* tumors, and the expression was enriched in the nucleus (Figure 5C). On the heatmap of RNA-Seq analyses, the enrichment of Shh pathway components was at a much higher degree in *Ptch1^–/+^;*3nSD MBs compared with *Ptch1^–/+^* MBs (Figure 5D), and Kyoto Encyclopedia of Genes and Genomes (KEGG; https://www.genome.jp/kegg/) analyses corroborated this notion (Figure 5E). These data unequivocally demonstrated that high-level Sufu promotes MB tumorigenesis at least in *Ptch1^–/+^* mice.

## Discussion

Although SHH was long regarded as a morphogen whose concentration gradient provides positional cues that direct tissue patterning, recent studies point to a transient triggering model for specifying digit identity (56). SUFU interacts with all 5 GLI activators and repressors produced through translation and posttranslational proteolytic processing, thereby playing a critical role in setting up the transcriptional interpretation of the SHH morphogenic gradient. Here, we observed that increased Sufu level allowed more Gli proteins to accumulate accordingly. This stabilizing effect of Sufu has been long noted in species ranging from fruit flies to mammals (17, 33, 57). By recruiting corepressors such as Sin3A to GLIs (58, 59), SUFU per se negatively regulates transcriptional responses of SHH signaling; however, by binding and stabilizing full-length GLI2 and GLI3, it can also negatively regulate SHH signaling through facilitating the production of truncated GLI repressors. The latter is likely the basis for the de-repression mechanism that accounts for the overactivated SHH pathway in SUFU-deficient animal models (19, 20). On the other hand, the same stabilizing activity also helps to produce the full-length GLI activators and, together with the propensity to accompany GLIs into the nucleus, renders SUFU a positive regulator of SHH signaling.

During limb development, Shh/Gli3 signaling along the anterior-posterior axis plays a critical role in assigning digit identity (42, 60). It has been reported that complete loss of Shh leads phalangeal bones to fuse into a single digit, whereas complete loss of Gli3 results in supernumerary digits without clear identity (60). On the other hand, mutant mice that expressed only the full-length Gli3^A^ exhibit duplication of digit 1, whereas mice that express only truncated Gli3^R^ repressor are postaxial polydactyly (61). In our 4nSD mice, elevated levels of Sufu led to accumulation of high levels of truncated Gli3^R^ repressors and the receding posterior Gli1 and Ptch1 domains (Figure 3, A and B), which were clear signs of enhanced Gli3^R^ repressive function. However, elevated Sufu also led to accumulation of full-length Gli3^A^ activator in the posterior limb bud region and the pathway target Gli1 (Figure 3, A and B), which could be attributed to both the stabilizing effect of Sufu as well as activation of Shh signaling. Since Shh signaling was clearly increased in receiving cells (Figure 3, B–D), the preaxial polydactylous phenotypes of Sufu gene copy number mutants are likely the net outcome of these two opposing forces in the limb bud. In postnatal cerebella of mouse mutants with increased *Sufu* gene dose, the same heightened Shh sensitivity was the force that powered the expansion of the EGL and the accelerated onset of foliation and sustained Shh signaling hampered GCPs from exiting the cycling phase (Supplemental Figure 3). Nevertheless, because elevated Sufu stabilized all forms of Gli proteins while likely leaving intact other Shh signaling regulatory mechanisms, cerebellar development eventually returned to normal.

Previously, we demonstrated the essential role of Sufu in MB formation initiated by heterozygotic loss of *Ptch1* in mice (18). Our current data regarding *Sufu* copy number mutants corroborated that finding by showing the opposite is also true, namely elevated Sufu promotes MB tumorigenesis. More strikingly, our analyses revealed the correlation of high-level SUFU expression with that of GLI1 in human MB samples, indicating that the positive role of SUFU also applies to human disease. Notwithstanding, the tumor-promoting role of SUFU revealed in our studies does not run contradictory to its status as a tumor suppressor, as the SHH pathway could be activated by way of derepression in MBs with *SUFU* mutations. This is because, in the absence of *SUFU*, truncated GLI repressors are not produced, thereby allowing SHH target genes to be activated. However, elevated SUFU permits the generation of more GLI activators, thereby rendering the SHH pathway to be activated to higher levels and exacerbating the consequence of the original tumor-driving mutations. Thus, this unorthodox tumor-promoting function of SUFU is no more than the flip side of the same coin that we called a novel type of molecular chaperone (17), and its revelation clarifies potentially counterintuitive clinical observations of high SUFU expression in MBs and ultimately may even pave way for novel strategies to reduce or reverse MB progression using proteolysis-targeting chimeric–based (PROTAC-based) approaches.

## Methods

### Animals and reagents.

Transgenic Gt(Rosa)26Sor alleles with hSUFU cDNA insertions were generated using the plasmid pRosa26-PA (pBigT) vector. Briefly, hSUFU cDNAs were cloned into the pBigT vector that contains a loxP-flanked cassette with a PGK-neo selectable marker and a tpA transcriptional stop sequence. The plasmid was then electroporated into ES cells and inserted into the Gt(Rosa)26Sor locus through homologous recombination. Correctly targeted mouse strains B6;129-Gt(ROSA)26Sor^tm(SUFU)^ (R26R-SUFU^WT^), B6;129-Gt(ROSA)26Sor^tm(SUFU*S342/6A)^ (R26R-SUFU^S342/6A^), and B6;129-Gt(ROSA)26Sor^tm(SUFU*S342/6D)^ (R26R-SUFU^S342/6D^) were maintained in a mixed C57 and 129/S6 outbred background. B6;129-*Ptch1^tm1Mps^* mice were originally obtained from The Jackson Laboratory (strain 003081), and *Ddx4*-Cre mice were purchased from the Animal Core Facility of Nanjing Medical University. Mice of both male and female sexes were utilized in all experiments, as no discernible sex differences were noted in any of the measured endpoints. To stage the embryos, the morning of the day on which a vaginal plug was observed was defined as E0.5, and the day of birth was designated as P0. To genotype animals, genomic DNA was prepared by incubating tail snips in 100 mL lysis buffer (50 mM Tris-HCl, pH 8.8, 1 mM EDTA, and 0.5% Tween 20) supplemented with proteinase K (25 mg/mL) at 56°C overnight. PCR primers used for genotyping and other experiments, antibodies, and other key reagents are listed in Supplemental Tables 4 and 5.

### Bone and cartilage staining.

To prepare for bone and cartilage staining, whole embryos were immersed in hot water (65°C–70°C) for 30 seconds before peeling off the skin and to be eviscerated. The skeletons were then preserved in 95% ethanol. A cartilage-specific Alcian Blue solution (Sigma-Aldrich) containing 150 mg/L Alcian Blue 8GX in 75% ethanol and 20% acetic acid was used to stain embryos for 24 hours in dark. Embryos were then cleaned with 95% ethanol and stained for an additional 24 hours with fresh Alcian Blue. The embryos were then stained in a bone-specific Alizarin Red solution (Sigma-Aldrich) containing 50 mg Alizarin Red per liter of 2% potassium hydroxide for 3–8 hours. Caution needs to be taken to preserve the specimen’s integrity and avoid unwanted corrosion by KOH. After gentle washing with 1% KOH, the specimens were photographed (62).

### RNA in situ hybridization.

Whole-mount RNA in situ hybridization was performed on E11.5 embryos with digoxigenin-labeled antisense probes and a chromogenic substrate for alkaline phosphatase (BM purple, Roche) specifically designed for precipitating enzyme immunoassays. The detailed procedure was as previously described (63).

### IHC and IF staining.

Early postnatal (P0 and P7) and adult (P28 and 6 months) mouse cerebella were dissected and immersed in 4% paraformaldehyde solution at 4°C for 24 hours. Cerebella after P7 were collected following intracardiac perfusion. Cerebellar tissues were embedded in paraffin according to standard methods and sectioned at 5 μm. Midsagittal sections were used for H&E staining. The MB micro tissue arrays were obtained from the Nanjing Drum Tower hospital, and fresh MB tissue sections were obtained from the Nanjing Children’s Hospital. Heat-induced antigen retrieval for IF and IHC staining was carried out in 10 mM sodium citrate buffer (pH 6.0) at 95°C. Goat anti-rabbit IgG H&L (HRP) was used as the secondary antibody for IHC staining, and Alexa Fluor 488– or Alexa Fluor 594–labeled donkey anti-rabbit IgG (H+L) was used for IF staining (Supplemental Table 5). Results from both IHC and IF staining were analyzed and scored using ImageJ software (NIH). In semiquantification of the MB array, staining intensity scoring was performed by an experienced pathologist reader, and percentage of positive cell ratio scores was determined based on the following matrix: 0, under 5%; 1, 6%–25%; 2, 26%–50%; 3, 51%–75%; and 4, over 76%. IHC scores were calculated as the products of intensity and percentage scores.

### Isolation of primary MEFs.

To isolate MEFs, torsos of E13.5– E14.5 embryos were separated from heads and visceral organs, cut into small pieces, and trypsinized in 10 cm petri dishes for 5–10 minutes. The dissociated cells were then plated out and allowed to grow into monolayer culture. After this expansion step, MEFs were frozen in aliquots and stored in liquid nitrogen.

### Cell culture, Western analyses, and protein half-life measurement.

Primary MEFs were cultured in DMEM supplemented with 10% FBS, 1×glutamine, 1 mM sodium pyruvate, 1% penicillin, and streptomycin at 37°C with 5% CO_2._ Biologically active murine Shh-conditioned medium (ShhN-CM) and control medium (293T-CM) were produced as described previously (17). To quantify protein half-lives, primary MEFs were grown to 90% confluence and then treated with ShhN-CM or control medium for 24 hours before adding CHX (10 μM) for durations as indicated. Cells were then washed with Dulbecco’s Phosphate-Buffered Saline (DPBS) and lysed in ice-cold RIPA lysis buffer. Each sample containing 50 μg lysate was separated on an 8% SDS/PAGE gel under denaturing conditions. Densitometric analysis was carried out using NIH ImageJ image analysis software. For other Western analysis experiments, cultured cells were washed twice with cold DPBS and subsequently lysed in ice-chilled RIPA lysis buffer (150 mM NaCl, 50 mM Tris-HCl, pH 7.5, 1 mM EDTA, pH 8.0, 0.5% sodium deoxycholate, 1% NP-40, 0.1% SDS, 2% sodium fluoride and 0.5% sodium orthovanadate supplemented with protease inhibitor cocktail) at 4°C for 30 minutes. After clarification by a brief centrifugation (14,000*g*, 20 minutes), the supernatant was transferred to a separate tube, and the protein concentration was determined by bicinchoninic acid assay. Each lysate with 1X loading buffer was denatured at 95°C for 5 minutes. Then, the lysates were resolved on 8% SDS/PAGE and blotted onto PVDF membranes. The films were blocked into 5% skimmed milk in 1× TBST and probed with indicated primary antibodies followed by horseradish peroxidase–conjugated secondary antibodies. Signals were visualized using Clarity Western ECL substrate. Antibodies used are listed in Supplemental Table 5.

### RNA extraction and quantitative real-time PCR analyses.

Total RNA was isolated from cultured cells using the RNAiso Plus reagent, and reverse transcription was carried out using HiScript II Q RT SuperMix from a qPCR kit (Vazyme). Real-time PCR was carried out using AceQ qPCR SYBR Green Master Mix (Vazyme) on a real-time PCR system (Roche), with primers as listed in Supplemental Table 4. Expression levels of indicated genes were normalized to the internal control, and relative expression levels were evaluated using the 2^−ΔΔCT^ method. Each target was measured in triplicates.

### RNA-Seq.

RNA-Seq was performed using RNAs obtained from the cerebellar or MB tissues in triple repeats. Sequence libraries were generated and sequenced by CapitalBio Technology. The differential expression gene (DEG) analyses were performed using the Limma package (64). The fold change cut-off of ≥1 was used for each DEG. *P* values were calculated for the means using the *t* test method. The significance level was set at 0.05 for FDR-corrected *P* values. Gene enrichment analysis for DEGs was performed using gene set enrichment analysis software (65, 66).

### Statistics.

Statistical analyses were performed using GraphPad Prism version 9.0. Data are presented as the mean ± SD. The differences among multiple treatment groups were analyzed with a 2-tailed unpaired Student’s *t* test or 1-way ANOVA and 2-way ANOVA. A *P* value less than 0.05 was regarded as statistically significant.

### Study approval.

All animal experiments were performed under protocols approved by the Animal Core Facility of Nanjing Medical University (no. 14030113-4). For studies involving humans, participants gave verbal informed consent or signed informed consent was obtained according to Nanjing Drum Hospital regulations.

### Data availability.

The sequencing data from this study have been deposited into the NCBI’s Gene Expression Omnibus database (GEO GSE248491). The public resources used for the data analysis were described in the original citation (7). All other data generated or analyzed during this study are presented in the manuscript and its supporting data values file. The underlying data generated in this study are available from the corresponding author upon request.

## Author contributions

SYC and CL conceived, designed, and obtained funding support for the study. BH, Yu Wang, and SY performed the experiments with assistance from YZ, LK, BG, Yue Wang, and ZZ. CL and TY supervised the study. XP and XW provided human MB samples, and CH provided ES cells with *hSUFU* insertions for generating transgenic mice. SYC, CL, and BH wrote the manuscript. The order of co–first authors was determined according to individual contributions to data acquisition, analyses, and manuscript preparation.

## Supplementary Material

Supplemental data

Unedited blot and gel images

Supporting data values

## Version 1 02/15/2024

In-Press Preview

## Version 2 03/22/2024

Electronic publication
