# Supplementary material for: Increasing Sufu gene dosage reveals its unorthodox role in promoting polydactyly and medulloblastoma tumorigenesis
Source: JCI Insight. 2024 Feb 15;9(6):e176044. doi: 10.1172/jci.insight.176044 (PMC10972619; doi:10.1172/jci.insight.176044)
Supplement: Supplemental data [file jciinsight-9-176044-s242.pdf]

Increasing *Sufu* Gene Dosage Reveals Its Unorthodoxical Role in Promoting Polydactyly and Medulloblastoma Tumorigenesis

Boang Han[1]*, Yu Wang[1]*, Shen Yue[1]*, Yun-hao Zhang[1], Lun Kuang[1], Bin-bin Gao[1], Yue Wang[1], Ziyu Zhang[2], Xiaohong Pu[3], Xin-fa Wang[4], Chi-chung Hui[5], Ting-ting Yu[1¶], Chen Liu[1¶], and Steven Y. Cheng[1¶]
1 Department of Medical Genetics, Nanjing Medical University, Nanjing 211166, China.
2 Key Laboratory of Women's Reproductive Health of Jiangxi, Jiangxi Maternal & Child Health Hospital, Nanchang, Jiangxi 330006, China.
3 Departments of Pathology, Nanjing Drum Tower Hospital, the Affiliated Hospital of Nanjing University Medical School, Nanjing 210008, China.
4 Department of Neurosurgery, Children's Hospital of Nanjing Medical University, Nanjing 210093, China.
5 Program in Developmental and Stem Cell Biology, the Hospital for Sick Children, and Department of Molecular Genetics, University of Toronto, Toronto, ON M5S 1A8, Canada

Supplementary Information

Supplementary Figures

sFigure 1

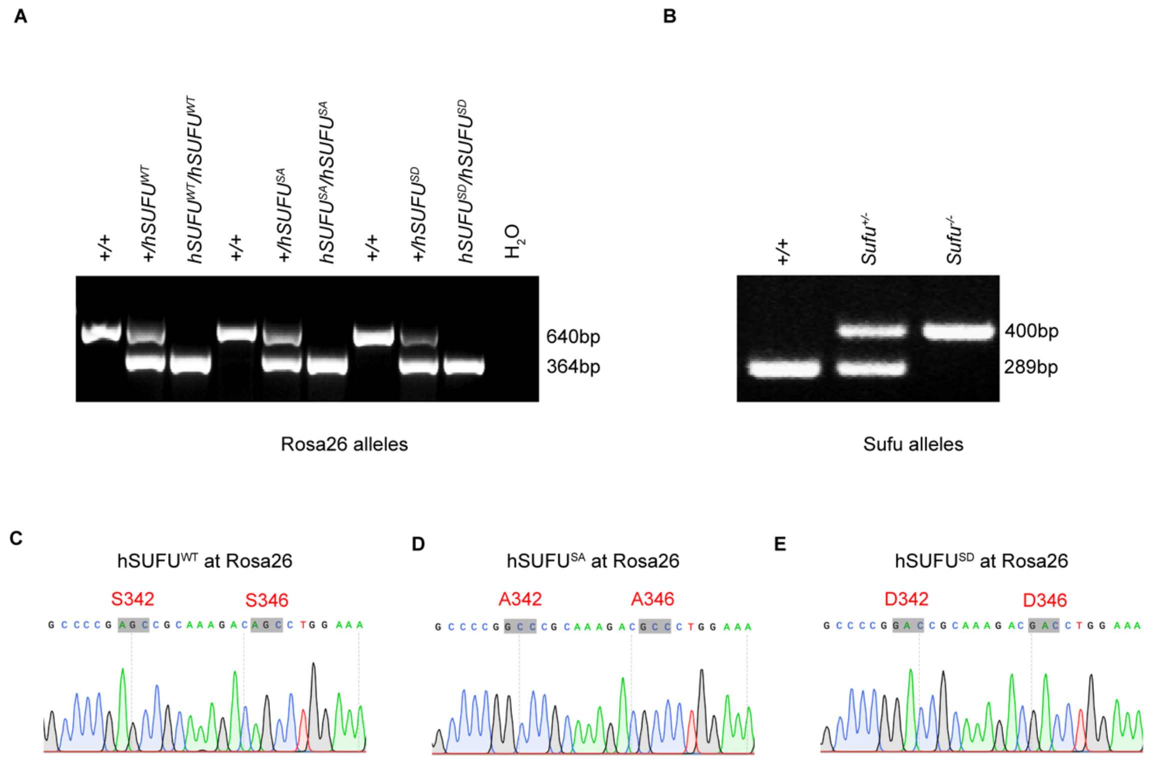

Supplementary Figure 1. Verification of *hSUFU* insertions at Rosa26 locus.

(A) PCR-based genotyping of *hSUFU* insertions at Rosa26 locus and (B) knock-out deletion of endogenous mouse *Sufu* gene. The PCR primers were described in Supplementary Table 4 and the amplified DNA fragments were resolved on 2.3% agarose gel. (C) Sequencing confirmation of inserted *hSUFU* alleles. WT, SA, and SD respectively refer to wild type or mutations at 342 and 346 positions.

sFigure 2

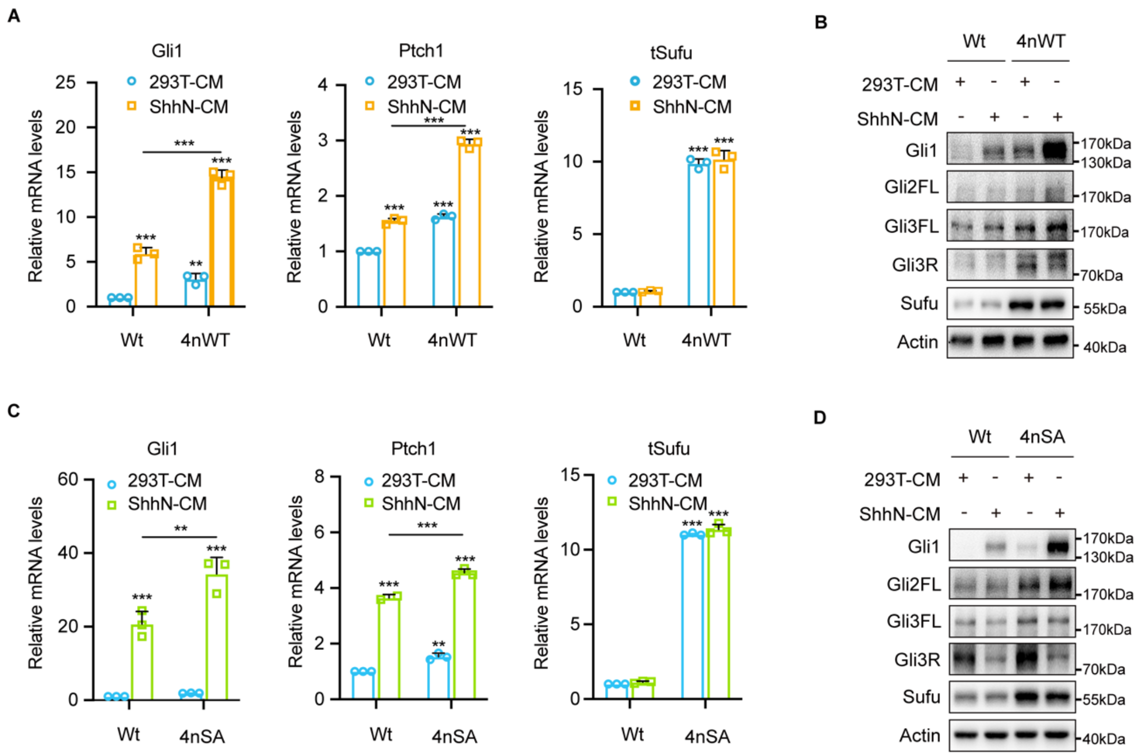

Supplementary Figure 2. Increasing *Sufu* gene dosage sensitizes receiving cells to Shh signaling.

(A) Real-time quantitative PCR detection of Shh signaling targets *Gli1* and *Ptch1* as well as *Sufu* in 4nWT MEFs (n=3). (B) Western analysis of various Gli and Sufu proteins in 4nWT MEFs (n=2). (C) Similar PCR (n=3) and (D) Western (n=3) analyses in 4nSA MEFs as shown in (A) and (B). Prior to harvesting, the MEFs were treated with ShhN or sham conditioned medium for 24 hours for to activate Shh signaling. Data represent mean ± SD and 2-way ANOVA with Tukey's multiple comparisons test was used in A and C. **P < 0.01; ***P < 0.001.

sFigure 3

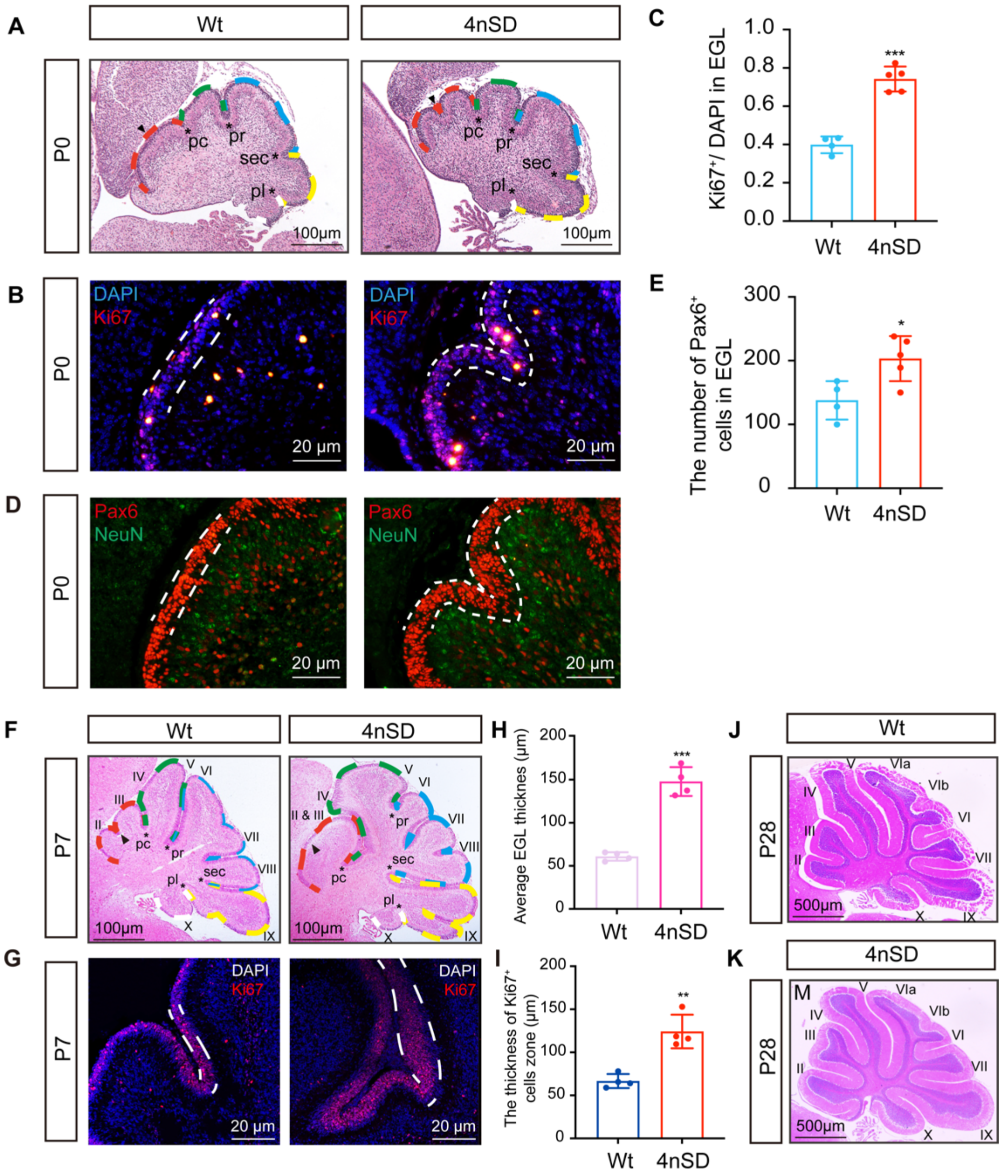

Supplementary Figure 3. High level of Sufu in 4nSD mice transiently accelerates cerebellar development

(A) At P0, SUFU$^{S342/6D}$ accelerated the fissure formation on the anterobasal cardinal lobe (red outline). It was suggested that SUFU$^{S342/6D}$ promoted the proliferation of GCPs (n=5). (B&C) The ratio of Ki67^{+} GCPs from the region was significantly increased in 4nSD samples (n=5). (D&E) 4nSD samples substantially had more Pax6^{+} GCPs from the area. (n=4). (H&I) At P7, the EGL thickness and the Ki67^{+} GCPs of the one lateral side from

the fissure pc (white dotted outline) in the 4nSD samples were thicker than the Wt samples (n=5). (J&K) The P28 4nSD mice showed no abnormalities in the cerebellum (n=5). Data represent mean ± SD and one-way ANOVA with Tukey's multiple comparisons test was used for statistical analysis in C, E, H and I. $^{*}P < 0.05$; $^{**}P < 0.01$; $^{***}P < 0.001$.

sFigure 4

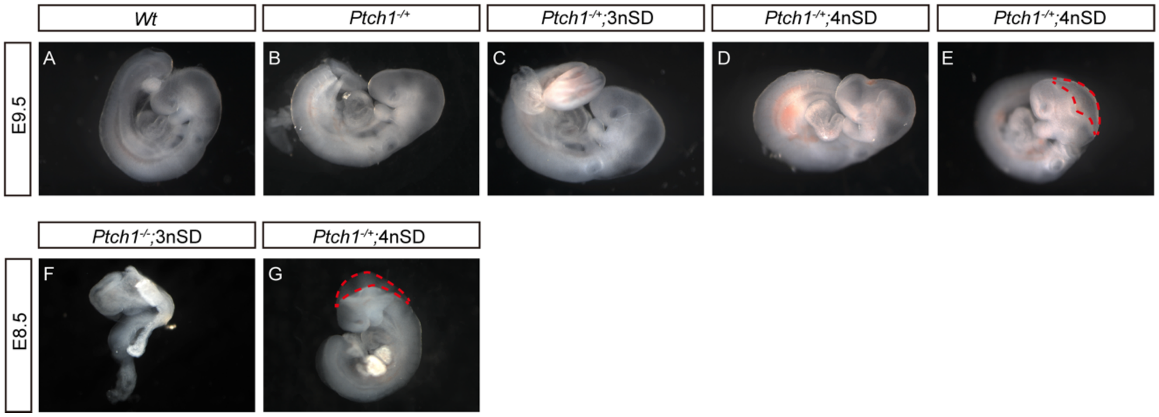

Supplementary Figure 4. The failure of the neural tube to close is what causes *Ptch1*$^{-/+}$;4nSD embryos to be lethal. Related to Figure 3.

(A-E) Embryos of various genotypes at E9.5 and (F-G) at E8.5. The unclosed *Ptch1*$^{-/+}$;4nSD neural tube is marked in red dashed line (n=3).

Supplementary Tables

Supplementary Table 1

IHC staining of GLI1 and SUFU

		GLI1				SUFU			
Sample NO.	Tumor type	Staining Intensity	percentage of cells stained (%)	Stained cell ratio score	IHC score	Staining Intensity	percentage of cells stained (%)	Stained cell ratio score	IHC score
1	MB	1	20	1	1	1	80	4	4
2	MB	1	5	0	0	1	80	4	4
3	Meningioma	0	0	0	0	1	20	1	1
4	MB	1	40	2	2	1	80	4	4
5	MB	1	10	1	1	0	0	0	0
6	MB	1	30	2	2	2	30	2	4
7	MB	1	15	1	1	1	40	2	2
8	MB	1	5	0	0	1	80	4	4
9	MB	1	10	1	1	2	20	1	2
10	MB	1	5	0	0	0	0	0	0
11	MB	1	10	1	1	1	90	4	4
12	MB	0	0	0	0	1	20	1	1
13	MB	2	20	1	2	0	0	0	0
14	MB	1	40	2	2	2	80	4	8
15	MB	1	70	3	3	2	80	4	8
16	MB	1	10	1	1	0	0	0	0
17	MB	2	20	1	2	1	90	4	4
18	MB	2	80	4	8	2	90	4	8
19	MB	1	10	1	1	0	0	0	0
20	MB	2	20	1	2	1	70	3	3
21	MB	2	20	1	2	1	30	2	2
22	MB	1	40	2	2	1	70	3	3
23	MB	1	20	1	1	2	80	4	8

24	MB	1	60	3	3	2	60	3	6
25	MB	2	40	2	4	1	80	4	4
26	MB	0	0	0	0	1	90	4	4
27	MB	2	60	3	6	2	40	2	4
28	MB	0	0	0	0	0	0	0	0
29	MB	1	20	1	1	1	80	4	4
30	MB	1	20	1	1	0	0	0	0
31	MB	1	60	3	3	2	80	4	8
32	MB	1	80	4	4	2	70	3	6
33	MB	1	80	4	4	2	90	4	8
34	MB	1	60	3	3	2	80	4	8
35	MB	2	80	4	8	1	90	4	4
36	MB	2	20	1	2	1	80	4	4
37	MB	0	0	0	0	0	0	0	0
38	Glioma	0	0	0	0	1	90	4	4
39	MB	1	10	1	1	0	0	0	0
40	MB	1	30	2	2	2	40	2	4
41	MB	1	40	2	2	2	90	4	8
42	MB	1	60	3	3	2	90	4	8
43	MB	2	80	4	8	2	80	4	8
44	MB	1	60	3	3	0	0	0	0
45	MB	2	90	4	8	2	90	4	8
46	MB	1	40	2	2	1	100	4	4
47	MB	1	20	1	1	1	100	4	4
48	MB	1	40	2	2	2	30	2	4
49	MB	1	20	1	1	2	90	4	8
50	MB	1	10	1	1	2	60	3	6
51	MB	2	80	4	8	2	90	4	8
52	Meningioma	1	80	4	4	1	70	3	3
53	Glioma	1	10	1	1	1	80	4	4

Supplementary Table 2

Mendelian inheritance of hSUFU insertions at Rosa26 locus

Sufu$^{+/+}$;Rosa26$^{+/WT}$ ⚥		
Genotype	Expected Ratio	Observed Ratio
Sufu$^{+/+}$	0.25	0.28
Sufu$^{+/+}$;*Rosa26*$^{+/WT}$	0.5	0.5
Sufu$^{+/+}$;*Rosa26*$^{WT/WT}$	0.25	0.22
	X2=0.54	P>0.05 *
		n=184

Sufu$^{+/-}$;Rosa26$^{+/SA}$ ⚥		
Genotype	Expected Ratio	Observed Ratio
Sufu$^{+/-}$	0.125	0.15
Sufu$^{+/+}$	0.0625	0.15
Sufu$^{-/-}$;*Rosa26*$^{+/SA}$	0.125	0.11
Sufu$^{+/-}$;*Rosa26*$^{+/SA}$	0.25	0.23
Sufu$^{+/+}$;*Rosa26*$^{+/SA}$	0.125	0.16
Sufu$^{-/-}$;*Rosa26*$^{SA/SA}$	0.0625	0.05
Sufu$^{+/-}$;*Rosa26*$^{SA/SA}$	0.125	0.08
Sufu$^{+/+}$;*Rosa26*$^{SA/SA}$	0.0625	0.07
	X2=10.04	P>0.05
		n=203

Sufu$^{+/-}$;Rosa26$^{+/SD}$ ⚥		
Genotype	Expected Ratio	Observed Ratio
Sufu$^{+/-}$	0.125	0.12
Sufu$^{+/+}$	0.0625	0.09
Sufu$^{-/-}$;*Rosa26*$^{+/SD}$	0.125	0.11
Sufu$^{+/-}$;*Rosa26*$^{+/SD}$	0.25	0.25
Sufu$^{+/+}$;*Rosa26*$^{+/SD}$	0.125	0.13
Sufu$^{-/-}$;*Rosa26*$^{SD/SD}$	0.0625	0.08
Sufu$^{+/-}$;*Rosa26*$^{SD/SD}$	0.125	0.16
Sufu$^{+/+}$;*Rosa26*$^{SD/SD}$	0.0625	0.06
	X2=3.27	P>0.05
		n=359

Supplementary Table 3

Pre-axial polydactyly incidence among hSUFU insertion transgenic lines

$Sufu^{+/+}$;$Rosa26^{+/WT}$ ⚥			
Genotype	Total	Polydactyly	Incidence(%)
Sufu$^{+/+}$	28	0	0.00
Sufu$^{+/+}$;*Rosa26*$^{+/WT}$	53	2	3.77
Sufu$^{+/+}$;*Rosa26*$^{WT/WT}$	21	10	47.62
			n=102

$Sufu^{+/-}$;$Rosa26^{+/SA}$ ⚥			
Genotype	Total	Polydactyly	Incidence(%)
Sufu$^{+/-}$	28	0	0.00
Sufu$^{+/+}$	51	0	0.00
Sufu$^{-/-}$;*Rosa26*$^{+/SA}$	7	0	0.00
Sufu$^{+/-}$;*Rosa26*$^{+/SA}$	39	0	0.00
Sufu$^{+/+}$;*Rosa26*$^{+/SA}$	55	1	1.82
Sufu$^{-/-}$;*Rosa26*$^{SA/SA}$	3	0	0.00
Sufu$^{+/-}$;*Rosa26*$^{SA/SA}$	12	1	8.33
Sufu$^{+/+}$;*Rosa26*$^{SA/SA}$	20	11	55.00
			n=215

$Sufu^{+/-}$;$Rosa26^{+/SD}$ ⚥			
Genotype	Total	Polydactyly	Incidence(%)
Sufu$^{+/-}$	44	3	6.80
Sufu$^{+/+}$	36	2	5.60
Sufu$^{-/-}$;*Rosa26*$^{+/SD}$	40	1	2.50
Sufu$^{+/-}$;*Rosa26*$^{+/SD}$	122	3	2.50
Sufu$^{+/+}$;*Rosa26*$^{+/SD}$	57	5	8.80
Sufu$^{-/-}$;*Rosa26*$^{SD/SD}$	30	4	13.30
Sufu$^{+/-}$;*Rosa26*$^{SD/SD}$	64	31	48.40
Sufu$^{+/+}$;*Rosa26*$^{SD/SD}$	38	29	76.30
			n=431

Supplementary Table 4

Genotyping and Quantitative Real-time PCR Primers

Genotyping PCR Primers	Sequence
**Rosa26-F8731**	CTTGCTCTCCCAAAGTCGCT
**Rosa26-R**	GCGGGAGAAATGGATATGAAGTA
**pBigT-R**	CGGCCTCGACTCTACGATAC
**Exon 4-Left**	CCTACCCTTTCCAGTGAAG
**Exon 4-Del**	GCTGAATTCTTGACTCACTG
**Neo-marker**	GTGTCAGTTTCATAGCCT
**Exon 8-Right**	CTGTTTGTACTCATGGTC
**LacZ-Forward**	GACACCAGACCAACTGGTAATGGTAGCGAC
**LacZ-Reverse**	GCATTGAGCTGGGTAATAAGCGTTGGCAAT

Quantitative Real-time PCR Primers

Gene	Sequence	
	sense（5'-3'）	antisense（5'-3'）
Gli1	CCAAGCCAACTTTATGTCAGGG	AGCCCGCTTCTTTGTTAATTTGA
Ptch1	AAAGAACTGCGGCAAGTTTTTG	CTTCTCCTATCTTCTGACGGGT
Sufu	GTCAAGTACTGGTTGGGTGG	CCTGTAAACTCATGGACTCTG
Actin	CATTGCTGACAGGATGCAGAAGG	TGCTGGAAGGTGGACAGTGAGG
18s	ACACGGACAGGATTGACAGATTGATAG	ACCAGACAAATCGCTCCACCAAC

Supplementary Table 5

Antibodies and Commercial kits

Antibodies	Source	Identifier	Application
Rabbit anti Gli1	CST,USA	2534	WB(1:1000)
Rabbit anti Gli1	NOVUS,USA	NB600-600	IHC(1:50)
Rabbit anti Gli1	HUABIO,China	3389	IHC(1:50)
Rabbit anti Gli2	NOVUS,USA	NB600-874	WB(0.1µg/mL),IHC(1:50)
Goat anti Gli3	R&D,USA	AF3690	WB(1µg/mL)
Rabbit anti SUFU	Proteintech,China	26759	WB(1:1000),IHC(1:100)
Rabbit anti Ki67	Abcam,USA	ab16667	IHC(1:200),IF(1:200)
Rabbit anti Pax6	Biolegend,USA	901302	IF(1:1000)
Mouse anti NeuN	Millipore,USA	MAB377	IF(1:50)
Rabbit anti Actin	Affinity,China	Ab-AF7018	WB(1:1000)
Rabbit anti GAPDH	Affinity,China	Ab-AF7021	WB(1:1000)
Alexa Fluor® 488 donkey anti-mouse IgG (H+L)	Thermo Scientific,USA	1975519	IF(1:200)
Alexa Fluor® 594 donkey anti-rabbit IgG (H+L)	Thermo Scientific,USA	2066086	IF(1:200)
AffiniPure Goat Anti-Rabbit IgG (H+L)	Jackson ImmunoResearch,USA	111–005–003	WB(1:5000)
AffiniPure Donkey Anti-Goat IgG (H+L)	Jackson ImmunoResearch,USA	705–005–003	WB(1:5000)
anti-Dig antibody	Roche,USA	11 093 274 910	ISH(1:5000)

Commercial kits	Source	Identifier	Application
HiScript II Q RT SuperMix for qPCR kit	Vazyme,China	R223-01	RT-PCR
AceQ qPCR SYBR Green Master Mix	Vazyme,China	Q111-02	Real-time PCR
Bicinchoninic acid (BCA) assay	Thermo Scientific,USA	23225	Protein quantitative

DAB Stain Assay Kit	ZSGB-BIO,China	ZLI-9019	IHC

Supplementary Table 6

Chemicals and Reagents

Chemicals and Reagents	Source	Identifier	Application
TRIzol	Takara,Japan	9109	RNA Extraction
Complete protease inhibitor cocktail	Roche,USA	#04693132001	Protease inhibitor
Cycloheximide(CHX)	Millipore,USA	239763	Protein synthesis inhibitor
2×Rapid Taq Master Mix	Vazyme,China	R223-01	Genotyping
Alcian Blue 8GX	Sigma	A3157	Bone Stain
Alizarin Red	Sigma	A5533	Bone Stain
Goat Serum	Beoytime,China	C0265	IHC,ISH
Donkey Serum	Solarbio	SL050	IF
DNaseI	Roche,USA	776785	ISH
proteinase K	Roche,USA	3115879	ISH
10x transcription buffer	Roche,USA	1465384	ISH
Dig labeling mix	Roche,USA	1277073	ISH
RNase inhibitor	Roche,USA	799025	ISH
Formamide	Gibco,USA	15515-026	ISH
tRNA	Sigma,USA	R7876	ISH
Heparin	Sigma,USA	H3149	ISH
Blocking Reagent	Roche,USA	1096176	ISH
Levamisol	Sigma,USA	L9756	ISH
RNaseA	Roche,USA	10109169001	ISH
T7 RNA polymerase	Roche,USA	881775	ISH
T3 RNA polymerase	Roche,USA	1031171	ISH
Sp6 RNA polymerase	Roche,USA	810274	ISH
BM purple	Roche,USA	11442074001	ISH
