# Supplementary material for: Increasing Sufu gene dosage reveals its unorthodox role in promoting polydactyly and medulloblastoma tumorigenesis
Source: JCI Insight. 2024 Feb 15;9(6):e176044. doi: 10.1172/jci.insight.176044 (PMC10972619; doi:10.1172/jci.insight.176044)

Full unedited gel for Figure 3B

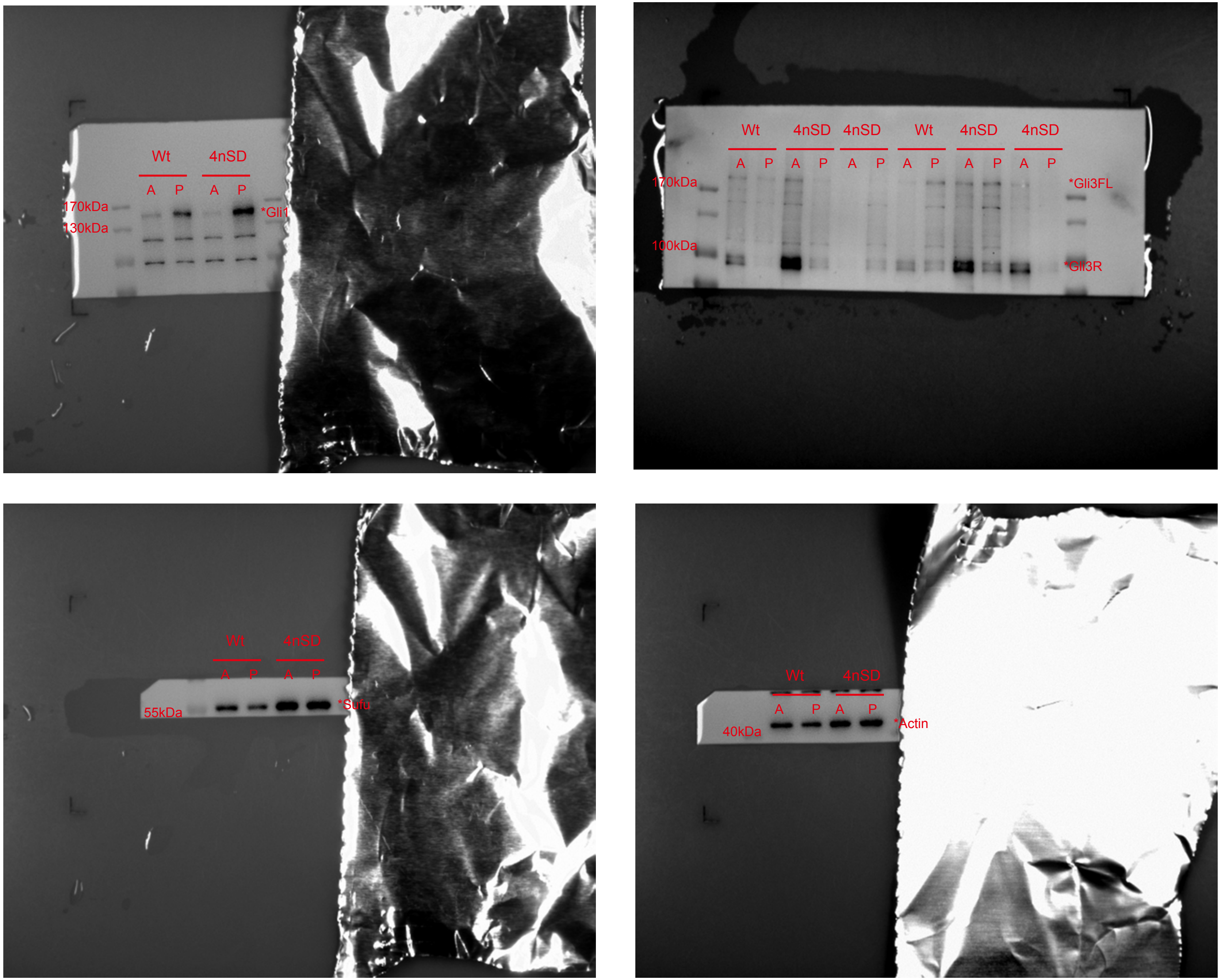

Full unedited gel for Figure 3D

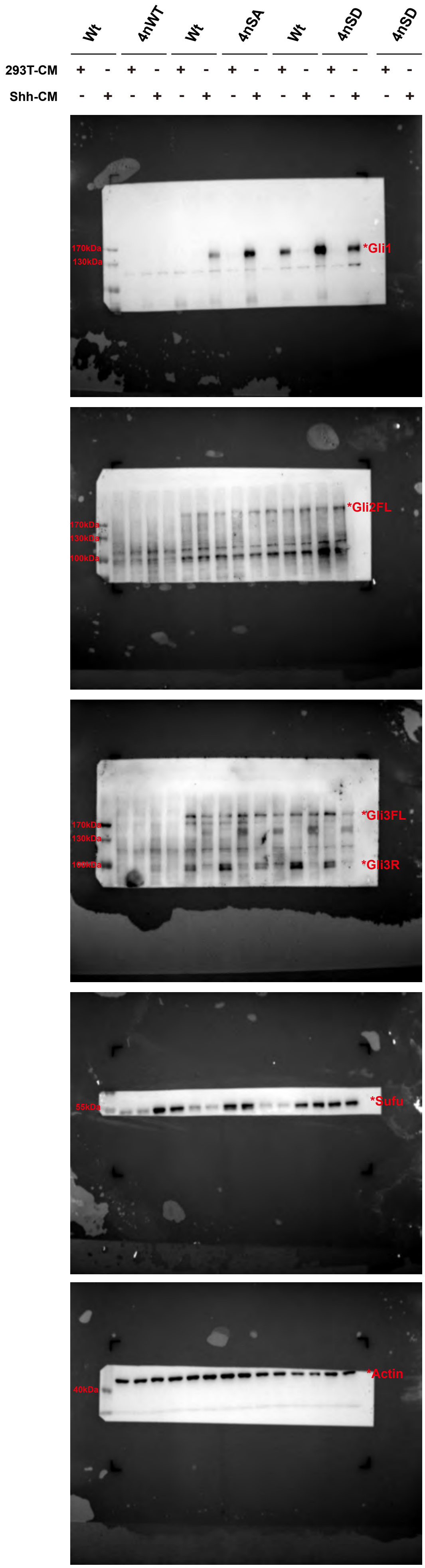

Full unedited gel for Figure 3E

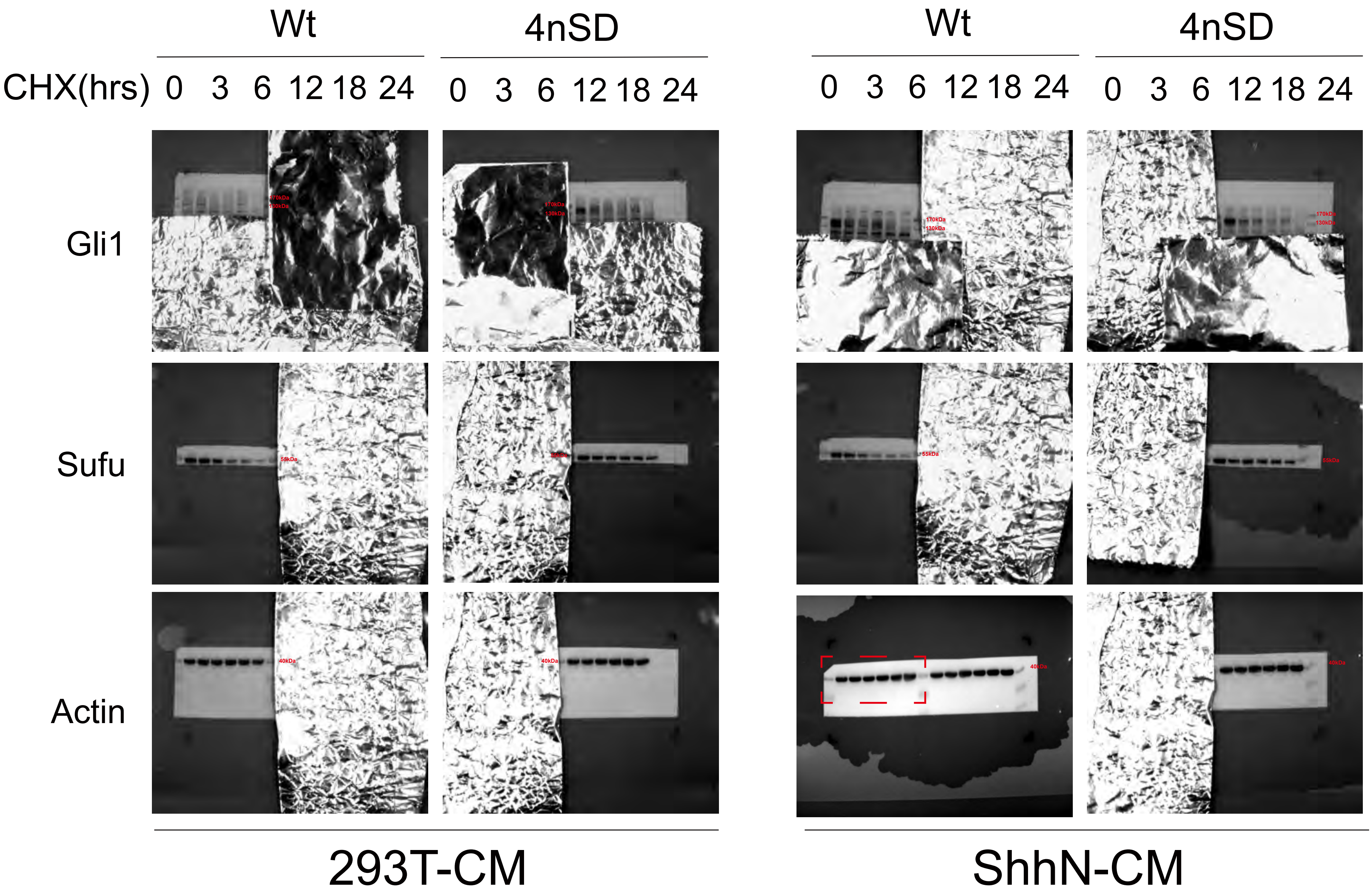

Full unedited gel for sFigure 2B

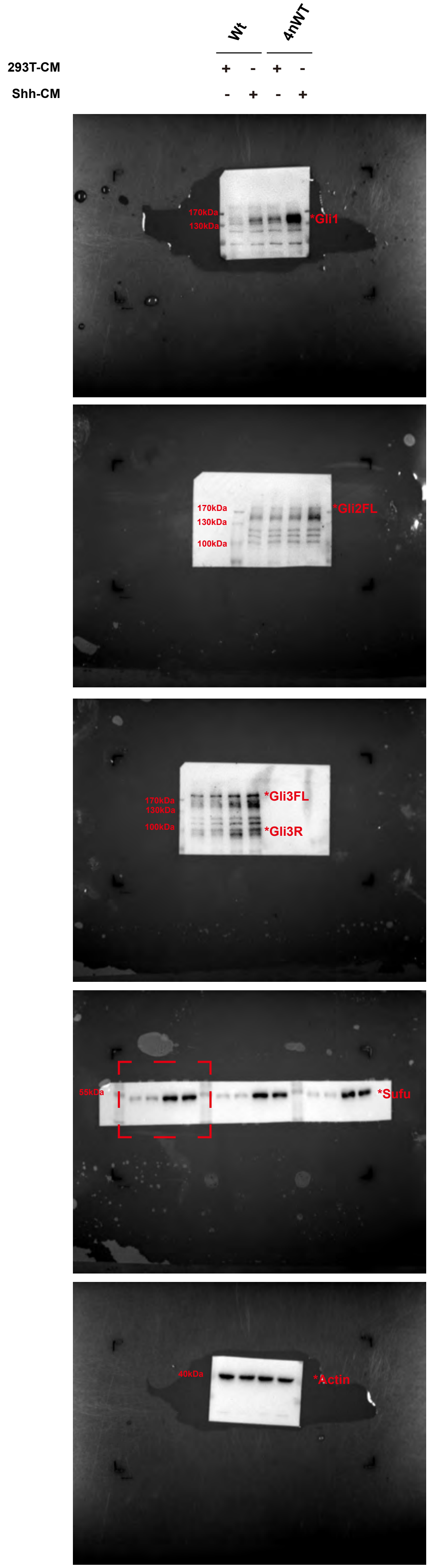

Full unedited gel for sFigure 2D

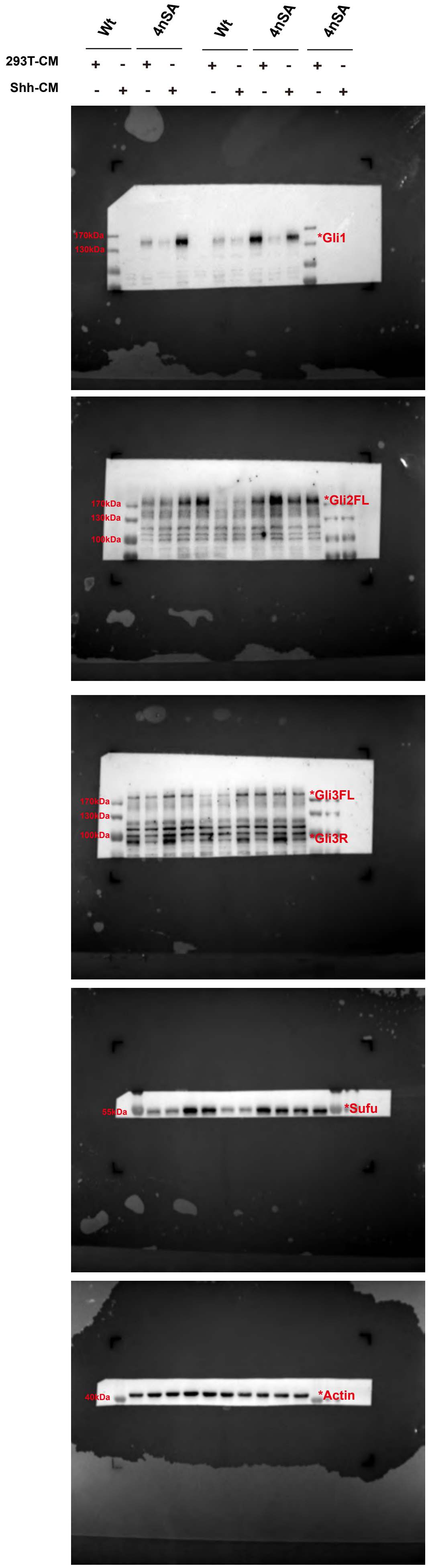

Supplement: Unedited blot and gel images [file jciinsight-9-176044-s243.pdf]
